# University of Botswana Public Health Medicine Unit contributions to the national COVID-19 response

**DOI:** 10.11604/pamj.2021.39.82.28314

**Published:** 2021-05-27

**Authors:** Keatlaretse Siamisang, Dineo Kebadiretse, Tiny Masupe

**Affiliations:** 1 Department of Family Medicine and Public Health, University of Botswana, Gaborone, Botswana,; 2 Department of Health Services Management, Ministry of Health and Wellness, Gaborone, Botswana

**Keywords:** University of Botswana, Public Health Medicine, response, COVID-19

## Abstract

COVID-19 was declared a Public Health Emergency of International Concern (PHEIC) in January 2020 and a pandemic in March 2020. Botswana reported its first case on 30^th^ March 2020 and as of 31^st^ January 2021 had 21,293 cases and 46 deaths. The University of Botswana Public Health Medicine Unit has made significant contributions to the national preparedness and response to COVID-19. The program alumni and Public Health Medicine residents have and continue to provide key technical support to the Ministry of Health and Wellness across the major pillars of COVID-19. This includes key roles in national and subnational coordination and planning, surveillance, case investigations and rapid response teams, points of entry, travel and transportation, infection prevention and control and case management. The unit is thus supporting the country in achieving the World Health Organization (WHO) primary objective of limiting human-to-human transmission, optimal care of the affected and maintaining essential services during the outbreak. The Public Health Medicine Unit has played a key role in capacity building including early rapid COVID-19 training of healthcare workers across the country. Furthermore faculty members and residents are involved in several COVID-19 research projects and collaborations.

## Commentary

COVID-19 was declared a Public Health Emergency of International Concern (PHEIC) under the International Health Regulations (IHR) on 30^th^ January 2020 and a pandemic in March 2020 [1,2]. The PHEIC declaration triggered an internationally coordinated response with the primary objective of limiting human-to-human transmission, caring for those affected and maintaining essential services [2]. As of 31^st^ January 2021, there were 21,293 cases in Botswana with 134 deaths [3].

Botswana has implemented several strategic measures in response to COVID-19. A national task force was set up to coordinate the national response. The Ministry of Health and Wellness (MOHW) initially conducted risk assessments of all the district health teams which then informed the response [4]. The country has been divided into nine COVID-19 zones, the largest of which is greater Gaborone, which has borne the brunt of the disease in the country. In an effort to minimize importation of the virus, the country's borders were closed on 24/03/2020 [5]. Additionally, port health systems were strengthened by capacitating ports of entry with testing services and quarantine sites for all returning travellers. To further improve early case detection within borders, there were strategies that emphasized on prevention and control measures. These included community sensitization and mobilization through public education via various media outlets. In addition, political leadership commitment enabled the community to easily adopt necessary measures such as handwashing, facemask use, and social distancing [4].

The Public Health Medicine (PHM) unit is part of the department of Family Medicine and Public Health in the Faculty of Medicine (FOM) at the University of Botswana (UB) with a mandate to train public health physicians through a 4-year residency programme, Master of Medicine (MMed) Public Health Medicine. Its involvement in the national preparedness and response began even before the first cases of COVID-19 were reported in Botswana and it continues to play a key role as the epidemic evolves. WHO under the International Health Regulations requests partners to support state members to achieve the objectives of a coordinated response [2]. The University of Botswana through its faculties and departments, has heeded this call and provided the support to the national COVID-19 response. This paper describes the specific support provided by the Public Health Medicine Unit to the national COVID-19 response.

**Technical Support for Ministry of Health and Wellness (MOHW):** from the onset of the epidemic, the PHM suspended formal didactic teaching and availed its trainees to the national response at national and sub-national levels. Alumni and residents of the programme all played important roles in the overall national coordination including among others, i) analysis of data and preparation of daily situational reports ii) residents played key roles within the national contact tracing, case management teams and testing and management of suspects at points of entry; iii) assessing and repurposing educational facilities for COVID-19 isolation and iv) substantial ongoing support to Greater Gaborone zone through case investigation, case management and testing. Residents' led case investigation teams played key roles in case mapping and identification of chains of transmission. More recently, the unit has had residents being part of the teams which assessed the capacity of the country's points of entry to handle a Public Health Emergency of International Concern.

**Capacity building:** the PHM has worked with MOHW to train healthcare workers in the districts. The unit has excellent relationships with the two mining hospitals in Botswana namely Jwaneng Mine Hospital and Orapa Mine Hospital. The hospitals leveraged on this relationship and requested the unit to train staff in the mining districts. Faculty members and residents conducted training on key COVID-19 topics including surveillance, infection prevention and control, case management and laboratory preparedness and response. Residents and staff also joined the Ministry of Health and Wellness teams in training other districts across the country. Much of this training was done even before the country reported its first case. Additionally, the unit was heavily involved in the weekly national virtual COVID-19 CME sessions. These sessions were organized by MOHW and were supported by local partners. The target audience was doctors and other healthcare workers both in the public and private sector. Public Health Medicine residents made key presentations including infection prevention and control and contact tracing. Furthermore, public health medicine residents supported greater Gaborone in training staff at local private hospitals on COVID-19 and technical support during outbreak investigation at one of the hospitals.

**Research:** the PHM unit is amongst the leaders in COVID-19 research in Botswana. Members are working with MOHW colleagues on the first few cases and contacts (FFX) investigation aimed at describing the clinical presentation and course of COVID-19, and estimating secondary infection and case fatality rates in Botswana. Faculty members and residents are also leading studies investigating the mental health impact of COVID-19 on healthcare workers. The aim of these studies is to determine the magnitude of stress, anxiety and depression among health care workers in the country, and also to shed light upon the protective and risk factors. The findings are expected to help guide interventions that could enable mitigation of mental health challenges during the COVID-19 pandemic in Botswana. Moreover, current literature on COVID-19 and health care workers mental health issues will be augmented. Public Health Medicine Residents are leading COVID-19 outbreak investigations in hospitals including infection prevention and control audits. This includes an outbreak investigation in the largest referral hospital in the country's public health system. This investigation included review of the hospital case reports and an assessment and audit of the hospital structures and processes with the aim of describing the cases in terms of person, place and time, identifying possible sources of infection and factors associated with continued transmission. Furthermore, the weekly journal clubs hosted in the Unit have strongly focused on COVID-19 research in recent months facilitating the sharing of up-to-date COVID-19 information. Undergraduate medical students have been participating in these journal clubs which has allowed faculty members and residents to directly share expertise with them.

**Safe campus and safe workplaces:** when initial restrictions of extreme social distancing were lifted and people returned to work, the PHM unit developed guidelines for safe return to work including guidance on development of workplace preparedness and response plans. These would facilitate implementation of infection and control measures, allow prompt isolation of suspects and enforce effective workplace controls. The unit also led the development of the university´s COVID-19 preparedness procedures and protocols and provided technical advice to the faculty on preventing workplace transmission of COVID-19.

## Conclusion

The University of Botswana Public Health Medicine Unit under the Department of Family Medicine and Public Health in the Faculty of Medicine has made significant contributions to the Botswana national COVID-19 preparedness and response. Through active involvement in all the pillars described above, the unit has significantly contributed to minimizing importation of cases, prevention of human-to-human transmission and maintaining essential services.
